# Microglial functional alteration and increased diversity in the challenged brain: Insights into novel targets for intervention

**DOI:** 10.1016/j.bbih.2021.100301

**Published:** 2021-07-20

**Authors:** Marie-Ève Tremblay

**Affiliations:** a Axe Neurosciences, Centre de Recherche du CHU de Québec-Université Laval, Québec, QC, Canada; b Molecular Medicine Department, Université Laval, Québec City, QC, Canada; c Department of Neurology and Neurosurgery, McGill University, Montréal, QC, Canada; d Division of Medical Sciences, University of Victoria, Victoria, BC, Canada; e The Department of Biochemistry and Molecular Biology, The University of British Columbia, Vancouver, BC, Canada

**Keywords:** Microglia, Physiological roles, Diversity, Chronic stress, Diet, Sleep, Infection, Neurodevelopmental disorders, Neuropsychiatric disorders, Neurodegenerative diseases

## Abstract

Microglia are the resident immune cells of the central nervous system (CNS) parenchyma, which perform beneficial physiological roles across life. These immune cells actively maintain CNS health by clearing toxic debris and removing dysfunctional or degenerating cells. They also modify the wiring of neuronal circuits, by acting on the formation, modification, and elimination of synapses—the connections between neurons. Microglia furthermore recently emerged as highly diverse cells comprising several structural and functional states, indicating a far more critical involvement in orchestrating brain development, plasticity, behaviour, and cognition. Various environmental factors, together with the individual genetic predispositions, confer an increased risk for neurodevelopmental and neuropsychiatric disorders, as well as neurodegenerative diseases that include autism spectrum disorders, schizophrenia, major depressive disorder, and Alzheimer's disease, across life. Microglia are highly sensitive to chronic psychological stress, inadequate diet, viral/bacterial infection, pollution, and insufficient or altered sleep, especially during critical developmental periods, but also throughout life. These environmental challenges can compromise microglial physiological functions, resulting notably in defective neuronal circuit wiring, altered brain functional connectivity, and the onset of behavioral deficits into adolescence, adulthood, and aging. This short review provides a historical and technical perspective, notably focused on my contribution to the field, on how environmental challenges affect microglia, particularly their physiological functions, and increase their diversity, which provides novel targets for intervention.

## Highlights

• Microglia perform beneficial physiological roles across life.
• Environmental risk factors can compromise these beneficial physiological roles.
• Microglia are indeed associated with most if not all brain disorders and diseases.
• Unraveling microglial functional diversity can provide novel targets for intervention.
• Dark microglia are an emerging subset that warrants functional investigation.

## 1 Introduction: microglial physiological roles

We are experiencing an important paradigm shift in the microglia field. Microglia are not neurotoxic cells that should be eradicated (Hellwig et al., 2013), instead they are highly beneficial immune cells that actively contribute, through their many physiological roles, to maintaining health. Over the past decade, the beneficial roles of microglia in the healthy brain have started to unravel (Tay et al., 2017b; Tremblay et al., 2011; Tremblay and Sierra, 2014).

As shown by a Pubmed search with microglia in all fields or in the title, there were only few papers published on the topic before the 1990s, and then a steady increase until the beginning of our century, followed by an exponential growth of microglial research (Tremblay et al., 2015). There is a first inflexion point in 2005, with the seminal discovery using non-invasive two-photon *in vivo* imaging that microglia—which used to be called ‘resting’ or ‘quiescent’ in the healthy brain, are extremely dynamic, continuously surveying the parenchyma with their highly motile processes (Davalos et al., 2005; Nimmerjahn et al., 2005). The development of non-invasive methods was a necessary condition for our understanding of the roles of microglia in the healthy brain (Tremblay, 2011; Tremblay et al., 2011). There is a second inflexion point in 2010, coincident with the discovery of their exclusive origin from the embryonic yolk sac (Ginhoux et al., 2010; Gomez Perdiguero et al., 2013). Microglia infiltrate the brain during embryonic development and they stay there throughout life, maintaining themselves through local self-renewal (Ajami et al., 2007; Huang et al., 2018; Tay et al., 2017b). The other immune cells that can transit through the brain, in different contexts of health and disease, instead come from the bone marrow (Mildner et al., 2007; Simard et al., 2006; Wohleb et al., 2013).

Also, in 2010, my postdoctoral work revealed that microglia respond to the changes in sensory experience during normal physiological conditions. For instance, a reduced microglial surveillance was observed in the mouse visual cortex after dark adaptation, induced by housing the animals in complete darkness for one week, from the beginning to the peak of the critical period for visual plasticity, and found to be reversed by re-exposure to circadian light for two days (Tremblay et al., 2010). *In vivo*, dynamic interactions between microglial processes and synaptic structures (axon terminals, dendritic spines) were frequently observed in the somatosensory and visual cortices (Tremblay et al., 2010; Wake et al., 2009). A combination of two-photon *in vivo* imaging and electron microscopy with immunostaining also revealed, in a non-invasive manner, that microglia frequently interact with pre-synaptic axon terminals, synaptic clefts, post-synaptic dendritic spines, and perisynaptic astrocytic processes during normal physiological conditions, increasing their contacts (phagocytic and non-phagocytic) with pre- and post-synaptic elements during dark adaptation and the subsequent re-exposure to circadian light (Tremblay et al., 2010). Chronic two-photon *in vivo* imaging over two days further revealed that dendritic spines contacted by microglia were more frequently eliminated than spines remaining non-contacted, which first suggested that microglia could be involved in their elimination (Tremblay et al., 2010).

Neuronal circuits are constantly refined, notably through the formation, modification, and elimination of synaptic structures, which allows for learning and memory, and adaptation to the ever-changing environment (Bernardinelli et al., 2014; Christoffel et al., 2011; Holtmaat and Svoboda, 2009). Based on ultrastructural analyses, different modes of microglia-mediated synaptic elimination were identified, including (i) phagocytosis, which is the engulfment of synaptic elements followed by their digestion within endosomes that fuse with lysosomes, whether it is complete or partial (i.e., trogocytosis), (ii) remodeling of the extracellular matrix leading to the collapse of dendritic spines, (iii) exophagy or extracellular “digestion” of extracellular debris, lipoproteins, and synaptic elements, as well as (iv) synaptic stripping, which was first described using the facial nerve injury model, and refers to the physical separation of pre- and postsynaptic elements (i.e., neuronal cell bodies, dendrites, dendritic spines) by intervening microglial processes (Acharjee et al., 2018; Blinzinger and Kreutzberg, 1968; Haka et al., 2016; Kettenmann et al., 2013; Paolicelli et al., 2011; Schafer et al., 2012; Trapp et al., 2007; Tremblay et al, 2010, 2012; Tremblay and Majewska, 2011; Weinhard et al., 2018).

With these findings and several other studies, microglia are now considered to be crucial during development for the survival of newborn neurons and their progenitors, the clearance of apoptotic cells, the formation of dendritic spines via brain-derived neurotrophic factor (BDNF) signaling, and the pruning of less active synapses through the complement pathway, fractalkine signaling, the triggering-receptor expressed on myeloid cells 2 (TREM2), and the neuronal or synaptic exposure of phosphatidyl serine (Filipello et al., 2018; Gunner et al., 2019; Györffy et al., 2018; Linnartz et al., 2012; Miyamoto et al., 2016; Paolicelli et al., 2011; Parkhurst et al., 2013; Schafer et al., 2012; Scott-Hewitt et al., 2020; Sierra et al., 2010; Tay et al., 2017b; Tremblay and Sierra, 2014). In adulthood, microglia are important effectors of plasticity, contributing to neuronal circuit maintenance and remodeling, as well as learning and memory, and the adaptation to an enriched or stressful environment (Milior et al., 2016; Parkhurst et al., 2013; Tay et al., 2017b; Tremblay and Sierra, 2014). Microglia further play physiological roles in axonal myelination, blood-brain barrier maintenance, vascular remodeling, and blood flow regulation (Császár et al., 2021; Hughes and Appel, 2020; Joost et al., 2019). Over the course of aging and upon exposure to various environmental risk factors for disease across the lifespan, microglia become more diverse and altered in their crucial physiological functions (Stratoulias et al., 2019; Tay et al., 2017a).

## 2 Microglial response to environmental risk factors

The exposure to various environmental risk factors combined with the individual genetic vulnerabilities results in different neurodevelopmental and neuropsychiatric disorders, as well as neurodegenerative diseases, depending on the stage of life. These risk factors comprise chronic psychological stress, but also inadequate nutrition, infection, and insufficient or altered sleep (Garofalo et al., 2020; Hanamsagar and Bilbo, 2017; Knuesel et al., 2014; Tay et al., 2017a). Prenatal development is a particularly sensitive period. Maternal immune activation, notably caused by chronic stress and viral or bacterial infection, combined with later environmental challenges, cumulating over a lifetime, was shown in epidemiological studies to confer an important risk for (i) autism spectrum disorders during childhood, (ii) schizophrenia, major depressive disorder, and other mental illnesses during adolescence and into adulthood, as well as (iii) Alzheimer’ disease, Parkinson's disease, and other age-related sporadic or late-onset forms of neurodegenerative diseases with aging (Knuesel et al., 2014; Tay et al., 2017a). These disorders and diseases arising across life are similarly associated with microglial phenotypic transformation or reactivity (e.g., proliferation, changes in morphology, increased phagocytic ability, release of pro-inflammatory mediators) and compromised physiological functions (Tay et al., 2017a).

Early microglial dysfunction during embryonic development due to genetic vulnerabilities and environmental risk factors (such as chronic stress, inadequate nutrition, and infection), leading to disturbances of the gut-brain axis and exacerbated inflammation, can result in impaired neuronal functions and the emergence of neurodevelopmental disorders (Bordeleau et al., 2020; Cryan et al., 2019; Knuesel et al., 2014; Tay et al., 2017a). An aberrant release of pro-inflammatory cytokines and impaired, mistargeted or exacerbated synaptic pruning can affect the density, maturation and wiring of neurons, translating into permanent defects of mature neural networks (Tay et al., 2017a). The imbalance of excitation to inhibition and an altered functional connectivity between brain regions were associated with neurodevelopmental disorders that include autism spectrum disorders and schizophrenia, but also attention-deficit/hyperactivity disorder (Moreau et al., 2020; Sohal and Rubenstein, 2019). Various environmental challenges such as chronic psychological stress, inadequate nutrition, and infection, but also pollution, can prime microglia (Bordeleau et al., 2020; Hanamsagar and Bilbo, 2017). Primed microglia are more susceptible to subsequent environmental challenges, leading to an abnormal cytokine secretion or an aberrant synaptic pruning upon re-exposure to chronic stress, inadequate nutrition, bacterial/viral infection, and sleep disturbances, among other challenges, during later life (Picard et al., 2021). These microglia-mediated changes in brain structure and function can together lead to the emergence of psychiatric disorders, such as major depressive disorder and schizophrenia, at puberty or during adulthood (Tay et al., 2017a).

In addition, microglial dystrophy and dysfunction, and perhaps senescence, during aging can be accelerated by various environmental risk factors for neurodegenerative diseases, such as chronic stress (Bisht et al., 2018; Tay et al., 2017a). Aged microglia have a reduced capability to survey the brain parenchyma and maintain homeostasis, due to their slower response to injury and impairment of phagocytosis, resulting in an accumulation of intracellular and extracellular debris (Burns et al., 2020; Hefendehl et al., 2014; Tay et al., 2017a, 2017b; Tremblay et al., 2012). The beneficial microglial physiological functions can further become compromised along the aging trajectory, resulting in (i) synaptic loss, which is considered one of the best pathological correlates of cognitive decline across major depressive disorder, schizophrenia, cognitive aging, and neurodegenerative diseases (Henstridge et al., 2016, p.; Scheff et al., 1996; Šišková and Tremblay, 2013; Streit et al., 2021), but also in (ii) axonal demyelination, (iii) blood-brain barrier dysfunction, and vascular pathology, increasingly involved in brain disorders and diseases across the lifespan (Cuddapah et al., 2019; Degenhardt et al., 2020; Haruwaka et al., 2019; Iii et al., 2016; Ota et al., 2019; Ouellette et al., 2020; Poliani et al., 2015; Shi et al., 2015; Thériault and Rivest, 2016).

In animal models of neuropsychiatric disorder or neurodegenerative disease, either rodents or non-human primates, microglial modulation or depletion using inhibitors of colony-stimulating factor 1 receptor (CSF1R) (Elmore et al., 2014) revealed—depending on the context and stage of life: improved outcomes on cognition and social behaviour, learning-dependent synapse formation, locomotion, anxiety, risk assessment behaviour (in juveniles), as well as spatial memory and male sex behaviours (in adults), and neurodegenerative disease pathology (in aging) (Asai et al., 2015; Elmore et al., 2014; Olmos-Alonso et al., 2016; Spangenberg et al., 2016; Tay et al., 2017a; Torres et al., 2016; VanRyzin et al., 2016). In human, treatment with the antibiotic minocycline, which dampens inflammatory responses and normalizes microglial phagocytosis (Mattei et al., 2017), also reduced the rewarding effects of methamphetamine abuse, produced beneficial effects for major depressive disorder, when added to serotonin-selective reuptake inhibitors, as well as attenuated positive and negative symptoms in early schizophrenia, combined with antipsychotics (Comer et al., 2020; Husain et al., 2017; Hutchinson et al., 2008; Levkovitz et al., 2010; Miyaoka et al, 2008, 2012; Plane et al., 2010; Sofuoglu et al., 2011; Tay et al., 2017a) (Table 1).

**Table 1:** Main microglial targets for therapeutic intervention. Their known mechanisms of action, examples of outcomes in murine models and humans, as well as limitations are summarized.

Target	Mechanisms of action	Outcomes in murine models	Outcomes in humans	Limitations	References
Classical complement pathway inhibitors	Antagonists and blocking antibodies acting on C1q, C3, C5 or other elements of the complement cascade	Gene knockouts protect synapses from elimination and prevent cognitive decline in models of aging and Alzheimer's disease (amyloid deposition, Tau pathology)	Eculizumab (or Soliris™, an anti-C5 antibody) already approved by the US Food and Drug Administration	Peripheral side effects vary based on the target	(Alawieh et al., 2018; Carpanini et al., 2019; Dalakas et al., 2020; Lee et al., 2010; Tay et al., 2017a; Tenner et al., 2018; Vecchiarelli et al., 2021; Woodruff et al., 2006; Zelek et al., 2019)
	Modulate microglial state and their interactions with synapses	Antibodies reduce disease progression and increase survival in models of amyotrophic lateral sclerosis and Huntington's disease	Clinical trials for Guillain-Barre syndrome, amyotrophic lateral sclerosis and Huntington's are underway	Tight balance in complement activity required to prevent mistargeted synaptic loss while allowing for learning, memory and other cognitive functions	
		Reduce injury and improve recovery in model of stroke			
Fractalkine signaling inhibitors	Antagonists or blocking antibodies of CX3CR1	Gene knockouts delay microglial brain colonization, migration and surveillance	No current trial (clinicaltrials.gov) pertaining to microglia, although variants were linked to schizophrenia, amyotrophic lateral sclerosis, age-related macular degeneration, and Alzheimer's disease, among other conditions	Cleaved and soluble or membrane-bound fractalkine exert different roles, as notably shown in Alzheimer's disease pathology (mouse models)	(Arnoux and Audinat, 2015; Fuhrmann et al., 2010; Gunner et al., 2019; Lee et al, 2010, 2014; Paolicelli et al., 2014; Tay et al., 2017a; Voronova et al., 2017)
	Fractalkine signaling is a main mode of communication between neurons and microglia in the brain	Influence survival of developing neurons, maturation, activity and plasticity of developing and mature synapses (e.g., via synaptic pruning), brain functional connectivity, adult hippocampal neurogenesis, learning, memory and behavioral outcomes		CX3CR1 is not only expressed by microglia, also by peripheral myeloid cells and by subsets of oligodendrocyte precursor cells during early postnatal development in mice	
	Regulates various microglial physiological properties	Prevent responsiveness of the brain and behaviour to chronic psychological stress			
		Improve amyloid pathology, reduce neuronal loss, but worsen Tau pathology in models of Alzheimer's disease			
TREM2 agonists	Different compounds act on a receptor of the immunoglobulin superfamily that regulates microglial survival, proliferation, phagocytosis, and metabolic state	Gene knockouts impair developmental synaptic pruning	Variants linked to Alzheimer's disease	Outcomes of activating TREM2 on microglial physiological functions remain largely undetermined	(Deczkowska et al., 2018; Filipello et al., 2018; Keren-Shaul et al., 2017; Krasemann et al., 2017; Ulland et al., 2017; Vecchiarelli et al., 2021)
		Result in the emergence of some microglial subtypes with exacerbated activity in contexts of neurodegenerative disease pathology and neuronal apoptosis	Several clinical trials underway, notably in the contexts of Alzheimer's disease	Possible detrimental consequences on synaptic loss	
		Modulate phagocytic clearance of amyloid		Peripheral myeloid cells could also be affected	
		Regulate blood flow			
CSF1R inhibitors	Different compounds act on a receptor tyrosine kinase required for the development, maintenance, and proliferation of microglia	Long-term impact on cognition and social behaviour	Clinical trials underway, notably in contexts of amyotrophic lateral sclerosis, mild cognitive impairment, Alzheimer's disease, glioblastoma, and several other disease conditions	Microglial depletion renews part of the population	(Elmore et al., 2014; Green et al., 2020; Lei et al., 2020, 2020; 2020; Olmos-Alonso et al., 2016; Tay et al., 2017a; VanRyzin et al., 2016)
	Depending on the dose, can modulate or deplete microglia	Increase locomotion		(some subsets are resistant)	
		Decrease anxiety-like behaviour		Replacing living microglia by dead ones can influence astrocytes and prevents essential microglial physiological functions	
		Increase risk assessment behaviour		Peripheral immune cells are also affected	
		Reverse changes in spatial memory			
		Impair male sex behaviours			
		Reduce inflammatory responses			
		Delay Alzheimer's disease pathology without altering amyloid load			
		Supress Tau pathology			
Minocycline	A tetracycline derived antibiotic already used in the clinic, notably for the treatment of acne	Dampens microglial release of inflammatory mediators	Decreases reward effects of methamphetamine	Not microglia specific, also acts on peripheral myeloid cells, astrocytes, oligodendrocytes, neurons, and endothelial cells, among other cell types in the brain	(Hui et al., 2019; Mattei et al, 2014, 2017; Nettis et al., 2021; Sellgren et al., 2019; Tay et al., 2017a; Vecchiarelli et al., 2021)
	Crosses the blood-brain barrier	Normalizes phagocytosis	Beneficial effects for major depressive disorder when added to selective serotonin reuptake inhibitors		
	Its mechanisms of action on microglia remain largely undetermined	Beneficial outcomes in models of maternal immune activation and ventral neonatal hippocampal lesion (for schizophrenia)	Reduces positive and negative symptoms in early schizophrenia when added to treatment with antipsychotic		
			Minocycline treatment for acne is associated with a reduction in incident schizophrenia risk		

## 3 Microglial diversity revealing targets for intervention

Recent single-cell transcriptome analyses revealed that in the healthy brain, microglia are highly diverse during early development, they mature to adopt a surveillant state into adulthood, and diversify again over the course of aging (Almanzar et al., 2020; Hammond et al., 2019; Zheng et al., 2021), possibly based on their lifelong environmental influences. These findings suggest that the physiological roles of microglia in the healthy brain, particularly those exerted during early postnatal development, may rely on specialized subsets of microglial cells (Stratoulias et al., 2019). Microglial diversity along the lifespan also displays sex differences, paralleling brain disorders and diseases (Bordeleau et al., 2019; Delage et al., 2021; VanRyzin et al., 2020; Villa et al., 2018). For instance, male microglia are bigger, express higher levels of MHC class I and II, display enhanced response to adenosine triphosphate, as well as increased expression of pro-inflammatory NFκB pathway genes, in mice (Guneykaya et al., 2018). While microglial sex differences remain largely unclear, there were found to be driven by a combination of gonadal hormones and sex chromosomes (Bordeleau et al., 2019; Delage et al., 2021; Kodama and Gan, 2019; VanRyzin et al., 2020). In response to disease pathology, microglia further diversify, displaying various subsets (i.e., subtypes, which can also display different phenotypes or ‘states’ when exposed to challenges) that remain to be defined structurally and functionally (Stratoulias et al., 2019).

**Fig. 1:**
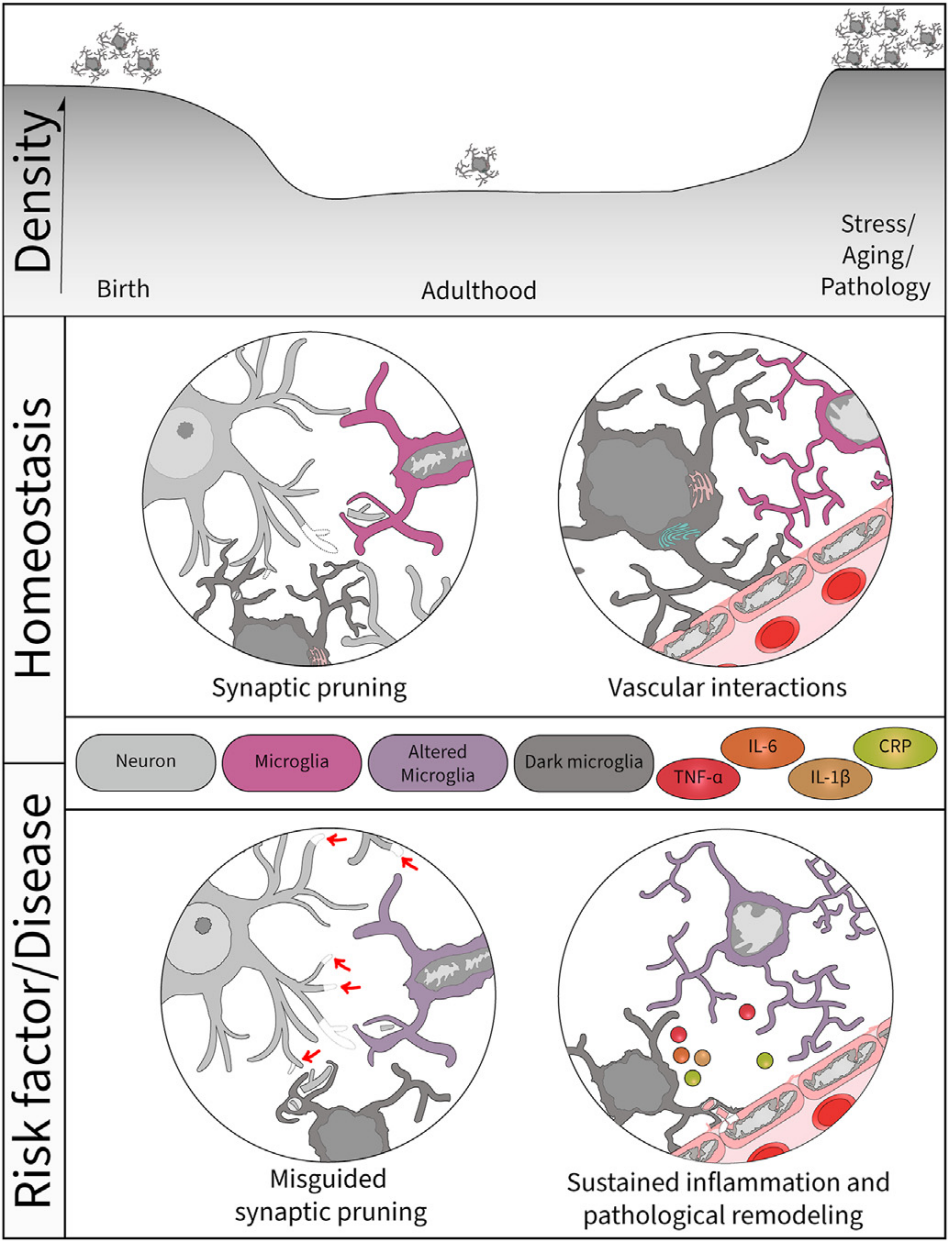
Summary of the knowledge surrounding dark microglia's roles in health and disease. Upper panel illustrating the density of dark microglia at different lifetime stages as seen in mice. In the middle panel, the homeostatic roles of microglia and suggested roles of dark microglia are shown, as able to perform synaptic pruning and synaptic stripping, but also contribute to the *glia limitans* during normal physiological conditions. Lower panel illustrates the current hypothesis that upon exposure to environmental risk factors and disease pathology, dark microglia become (i) involved in misguided synaptic pruning via exacerbated phagocytosis and extracellular digestion, (ii) contribute to the sustained inflammation within the brain parenchyma through their secretion of cytokines that may include TNFα, IL-6, IL-1β and CRP, similarly to the general microglial population (but to a greater extent), and (iii) take part in pathological vascular remodeling by endothelial cell phagocytosis, thus compromising the blood-brain barrier. TNFα ​= ​tumor necrosis factor alpha; IL-6 ​= ​interleukin 6; IL-1β ​= ​interleukin 1 beta; CRP=C-reactive protein.

Understanding how this microglial heterogeneity contributes to varied physiological and immune functions in health and disease will allow to design cellular interventions that specifically target (modulate, inhibit, or stimulate) contextually-relevant microglial functions (Stratoulias et al., 2019). While the biological relevance of putative microglial subsets inferred by transcriptomics remains elusive, using electron microscopy, my research team discovered in 2016 a distinct *bona fide* microglial subtype based on its unique properties and relationships with the vasculature and synapses (Bisht et al., 2016b). These pioneering studies identified ‘dark’ microglia as a specific microglial subset that is nearly absent from the brain of healthy young adult mice, and instead is significantly increased (up to 10-fold) in pathological states (including models of chronic stress-induced depression, aging, Alzheimer's and Huntington's pathology). These findings showed that dark microglia are strikingly different from the general microglial population at the ultrastructural level (Bisht et al., 2016b; Savage et al., 2020), even though the other (i.e., typical) microglia vary in their accumulation of intracellular debris and lipidic inclusions during aging (Tremblay et al., 2012). In fact, dark microglia (i) display unique markers of cellular stress (e.g., electron dense cytoplasm/nucleoplasm giving them a dark appearance in electron microscopy, dilation of the Golgi apparatus and endoplasmic reticulum) (Bisht et al., 2016a), (ii) have hyper-ramified processes that ensheath the vasculature, contributing to the *glia limitans* (i.e., glial end-feet layer of the blood-brain barrier), and (iii) extensively wrap around and engulf pre-synaptic and postsynaptic elements, as well as excitatory synaptic connections (Bisht et al., 2016b) (Fig. 1). These distinctive features support active, specialized roles of dark microglia in vascular and synaptic remodeling, as well as blood-brain barrier maintenance (St-Pierre et al., 2020).

Although dark microglia strongly downregulate canonical markers commonly used to identify microglia (e.g., CX3CR1, IBA1, P2RY12), which makes them difficult to observe with conventional light or fluorescent antibody-based techniques, we were able to uncover protein markers strongly expressed by dark microglia: (i) the microglia-specific 4D4, (ii) CD11b subunit of the complement receptor 3, and (iii) TREM2 (Bisht et al., 2016b), notably involved in phagocytosis and microglia-mediated synaptic pruning (Filipello et al., 2018; Schafer et al., 2012). These cells are encountered among several brain regions, including the hippocampus, amygdala, hypothalamus, striatum, and cerebral cortex (Bisht et al., 2016b; Hui et al., 2018; Savage et al., 2020). In addition, dark microglia are abundant, not only in adult pathological states, but also during normal postnatal development, particularly during the critical period when vascular networks and neuronal circuits are refined (St-Pierre et al., 2020). We also found that dark microglia's density significantly increases in adult mouse offspring exposed to maternal immune activation, using the viral mimic Poly I:C, especially in the males, which displayed the most pronounced schizophrenia-like behavioural deficits (Hui et al., 2018) (Fig. 1). Dark microglia are indeed present in the human brain, notably in the hippocampus, where they contact the vasculature, neurons and synapses, and in *post-mortem* brain samples of schizophrenia patients (St-Pierre et al., 2020).

From these original findings by my research group and others (St-Pierre et al., 2020), we hypothesize that dark microglia are a unique microglial subset that performs specialized functions in neuronal circuit and vascular remodeling, as well as blood-brain barrier maintenance. These functions would be exerted across normal postnatal development, maternal immune activation, stress-induced plasticity, cognitive aging, Alzheimer's and Huntington's disease pathology. They could be beneficial during normal development, but deleterious in the inflammatory contexts of maternal immune activation, chronic stress, aging, and neurodegenerative disease, where they may contribute to the pathological mechanisms (Fig. 1). Determining the molecular signature(s) of dark microglia, among other emerging microglial subtypes and states, is expected to provide unprecedented insight into their roles during normal development and their specific implication in the outcomes of various environmental risk factors for neurodevelopmental and neuropsychiatric disorders, as well as neurodegenerative diseases. In addition to the risk factors mentioned above, it would be particularly relevant from a translational perspective to further explore outcomes of the diet, urban and sedentary lifestyles, low socio-economic status, cigarette smoking and cannabis consumption. Longitudinal two-photon *in vivo* imaging approaches using selective molecular markers will be instrumental in determining whether dark microglia are a subtype with different intrinsic properties, which selectively becomes dark upon exposure to challenges to exert specialized functions, or whether the general microglial population can adopt a dark state. In addition, studying interactions between the peripheral immune cells infiltrating the CNS, which can also be affected by various environmental risk factors, and the microglial subtypes/states is an important topic of investigation, considering the therapeutic potential of these peripheral cells to replace or assist microglia with their essential functions within the brain parenchyma.

## 4 Perspectives and conclusion

Overall, these findings propose a new paradigm shift in the microglia field, from (i) a unique ‘multitasking’ cell type in the brain that responds to various environmental challenges, including infection, the diet and other lifestyle factors, as well as disease pathology, by transforming structurally and functionally, to (ii) a community of cells in which different subsets perform specialized physiological functions and respond differently to environmental challenges and disease pathology by adopting different states throughout life. Unraveling the influence of these challenges on these various emerging microglial subtypes and states, including the dark microglia, their specialized functions, and outcomes on disease pathology, promises to provide novel targets for symptomatic, disease-modifying, prevention or treatment strategies. These targets (see Table 1 for the main targets currently in clinical trial) are particularly expected to allow for the modulation, inhibition, or stimulation of contextually relevant microglial functions, across a wide range of neurodevelopmental and neuropsychiatric disorders, as well as neurodegenerative diseases in which microglia are significantly involved.

## Declaration of competing interest

The author declares no competing financial interests.
